# A Randomised, Placebo-Controlled, Crossover Study Investigating the Optimal Timing of a Caffeine-Containing Supplement for Exercise Performance

**DOI:** 10.1186/s40798-020-00246-x

**Published:** 2020-03-30

**Authors:** Andrew D. Davenport, Tom S. O. Jameson, Sean P. Kilroe, Alistair J. Monteyne, George F. Pavis, Benjamin T. Wall, Marlou L. Dirks, Nima Alamdari, Catherine R. Mikus, Francis B. Stephens

**Affiliations:** 1 University of Exeter, St Luke’s Campus, Heavitree Road, Exeter, EX1 2 LU UK; 2 Beachbody, LLC, 3301 Exposition Blvd, Santa Monica, CA 90404 USA

**Keywords:** Ergogenic aid, Metabolism, Paraxanthine, Performance, Perceived exertion, Sports nutrition

## Abstract

**Background:**

Pre-exercise supplements containing low doses of caffeine improve endurance exercise performance, but the most efficacious time for consumption before intense endurance exercise remains unclear, as does the contribution of caffeine metabolism.

**Methods:**

This study assessed the timing of a commercially available supplement containing 200 mg of caffeine, 1600 mg of β-alanine and 1000 mg of quercetin [Beachbody Performance Energize, Beachbody LLC, USA] on exercise performance, perception of effort and plasma caffeine metabolites. Thirteen cyclists (V̇O_2max_ 64.5 ± 1.4 ml kg^− 1^ min^− 1^ (± SEM)) completed four experimental visits consisting of 30 min of steady-state exercise on a cycle ergometer at 83 ± 1% V̇O_2max_ followed by a 15-min time trial, with perceived exertion measured regularly. On three of the visits, participants consumed caffeine either 35 min before steady-state exercise (PRE), at the onset of steady-state (ONS) or immediately before the time trial (DUR) phases, with a placebo consumed at the other two time points (i.e. three drinks per visit). The other visit (PLA) consisted of consuming the placebo supplement at all three time points. The placebo was taste-, colour- and calorie-matched.

**Results:**

Total work performed during the time trial in PRE was 5% greater than PLA (3.53 ± 0.14 vs. 3.36 ± 0.13 kJ kg^− 1^ body mass; *P* = 0.0025), but not ONS (3.44 ± 0.13 kJ kg^− 1^; *P* = 0.3619) or DUR (3.39 ± 0.13 kJ kg^− 1^; *P* = 0.925), which were similar to PLA. Perceived exertion was lowest during steady-state exercise in the PRE condition (*P* < 0.05), which coincided with elevated plasma paraxanthine in PRE only (*P* < 0.05).

**Conclusion:**

In summary, ingestion of a pre-exercise supplement containing 200 mg caffeine 35 min before exercise appeared optimal for improved performance in a subsequent fatiguing time trial, possibly by reducing the perception of effort. Whether this was due to increased circulating paraxanthine requires further investigation.

**Trial registration:**

**ClinicalTrials.Gov,** NCT02985606 **; 10/26/2016.**

## Key Points

- A commercially available supplement containing 200 mg of caffeine, 1600 mg of β-alanine and 1000 mg of quercetin [Beachbody Performance Energize, Beachbody LLC, USA] ingested 35 min before, but not immediately before or during, exercise reduced perceived exertion during 30-min cycling at 80% V̇O_2max_ and improved subsequent 15-min cycling time trial (TT) performance in trained cyclists by 5%.

- Plasma caffeine concentration was elevated during the TT with all supplement timing strategies, but paraxanthine was only elevated during the TT when the supplement was ingested 35 min before exercise, the only strategy to observe an ergogenic effect, suggesting a role for paraxanthine which requires further investigation. Caffeine and paraxanthine concentrations did not appear to be dependent upon *CYP1A2* genotype, a possible mediator of an individual’s response to caffeine ingestion.

- We suggest supplements containing low doses of caffeine (e.g. 200 mg) should be ingested around 60 min prior to the onset of expected hard exercise in order to reduce the perception of effort and maximize the improvement in performance. This is an improvement on the current broad recommendation of ingesting caffeine-containing supplements 60 min before exercise per se.

## Background

In efforts to optimize performance, the use of nutritional supplements is unsurprisingly widespread amongst elite athletes, with caffeine and multi-ingredient ‘pre-exercise drinks’ containing caffeine amongst some of the most commonly used [1, 2]. The ingestion of low doses of caffeine (≤ 3 mg kg body mass (bm)^− 1^) enhances endurance performance via its role as an adenosine receptor antagonist [3]. Indeed, during endurance exercise, adenosine blockade has been shown to reduce the perception of effort [4].

Despite vast amounts of research being undertaken over previous decades, little is currently known about the most efficacious time to ingest low doses of caffeine, particularly when consumed in a drink with other ingredients. Current guidelines typically recommend the ingestion of caffeine approximately 60 min prior to exercise [5]. This practice is reflected in research, with a recent meta-analysis observing that the vast majority of trials have been performed with caffeine ingested 60 min prior to a plethora of exercise scenarios [6]. It is, however, vital to recognize that the duration of the exercise bouts varies between studies, markedly changing the period of time between supplementation and the end of exercise. This is significant as caffeine, due to its effects on the central nervous system, is likely to be most ergogenic when perceived effort is increased, which will presumably be at its highest towards the end of exercise, and it appears to have a narrow window of action [6]. For example, it would appear that ingesting low-dose caffeine 60 min before the latter stages of a time trial (TT) would be more effective than earlier (> 80 min) or later (< 40 min) [6], but this is yet to be tested under identical experimental conditions.

The finding that the effects of ingesting caffeine tend to wane > 80 min prior to the end of a TT appears to be at odds with the fact that plasma caffeine concentrations remain elevated at a steady state for several hours after ingestion [7]. This is in line with another discrepancy in the caffeine literature around the mounting evidence of individual responses based on genotype [8]. Specifically, *CYP1A2* is the gene recently identified as a possible mediator of an individual’s response to caffeine ingestion. It encodes for cytochrome P450 1A2; an enzyme responsible for 95% of all caffeine metabolism [9], predominantly to paraxanthine (~ 79%), but also theobromine (~ 11%) and theophylline (~ 4%). Those with a homogenous A allele of the *CYP1A2* gene tend to produce more cytochrome P450, metabolize caffeine more quickly [10] and experience a significantly greater ergogenic effect of caffeine compared to those with a C allele in some [11, 12] but not all [13, 14] studies. However, if elevated plasma caffeine is the primary driver of observed ergogenic effects then, paradoxically, ‘slow’ metabolisers should see the greatest benefit. Thus, perhaps paraxanthine, a pharmacologically more potent adenosine-receptor antagonist that would be elevated sooner in ‘fast’ metabolisers, is responsible for the ergogenic properties of caffeine [15]. Indeed, in rats, paraxanthine, but not caffeine, also significantly increases extracellular dopamine levels in the dorsolateral striatum, increasing locomotor activation [16].

The aim of the present study was to investigate the effect on performance of timing of the ingestion of commercially available drink containing a low (200 mg, ~ 2.8 mg kg bm^− 1^) dose of caffeine, 1600 mg of β-alanine, 1000 mg of quercetin and 4.5 g sucrose [Beachbody Performance Energize, Beachbody LLC, USA] before a 15-min cycling TT in trained cyclists. It was hypothesised that the ingestion of caffeine 70 vs. 35 and 0 min prior to the TT would improve performance via a reduction in perceived exertion and correspond with greater circulating caffeine and paraxanthine.

## Methods

### Participants

Fourteen (12 male, 2 female) well-trained cyclists (all had competitive time trial and/or triathlon experience, and 9 of whom were competitive road or criterium racers) were recruited (age 28 ± 2 years, bm 71 ± 2 kg, height 175 ± 3 cm, V̇O_2max_ 64.5 ± 1.4 (range 58.2 to 71.2) mL kg bm^− 1^ min^− 1^, *W*_max_ 334 ± 10 W). Via the use of questionnaires to assess both daily caffeine intake, and pre-race caffeine strategies, it was ascertained that eleven of the participants were habitual caffeine users (≥ 50 mg day^− 1^), and 7 regularly used caffeine as part of their pre- and/or during race routines. Both female participants were taking a combined oral contraceptive pill for the duration of the study to negate for any potential effects of menstrual cycle stage on caffeine metabolism. Following an explanation of the experimental protocol and its risks, all individuals provided written, informed consent. The study was approved by the University of Exeter Sport and Health Sciences Ethics Committee (161,026/A/10).

### Study design

**Fig. 1:**
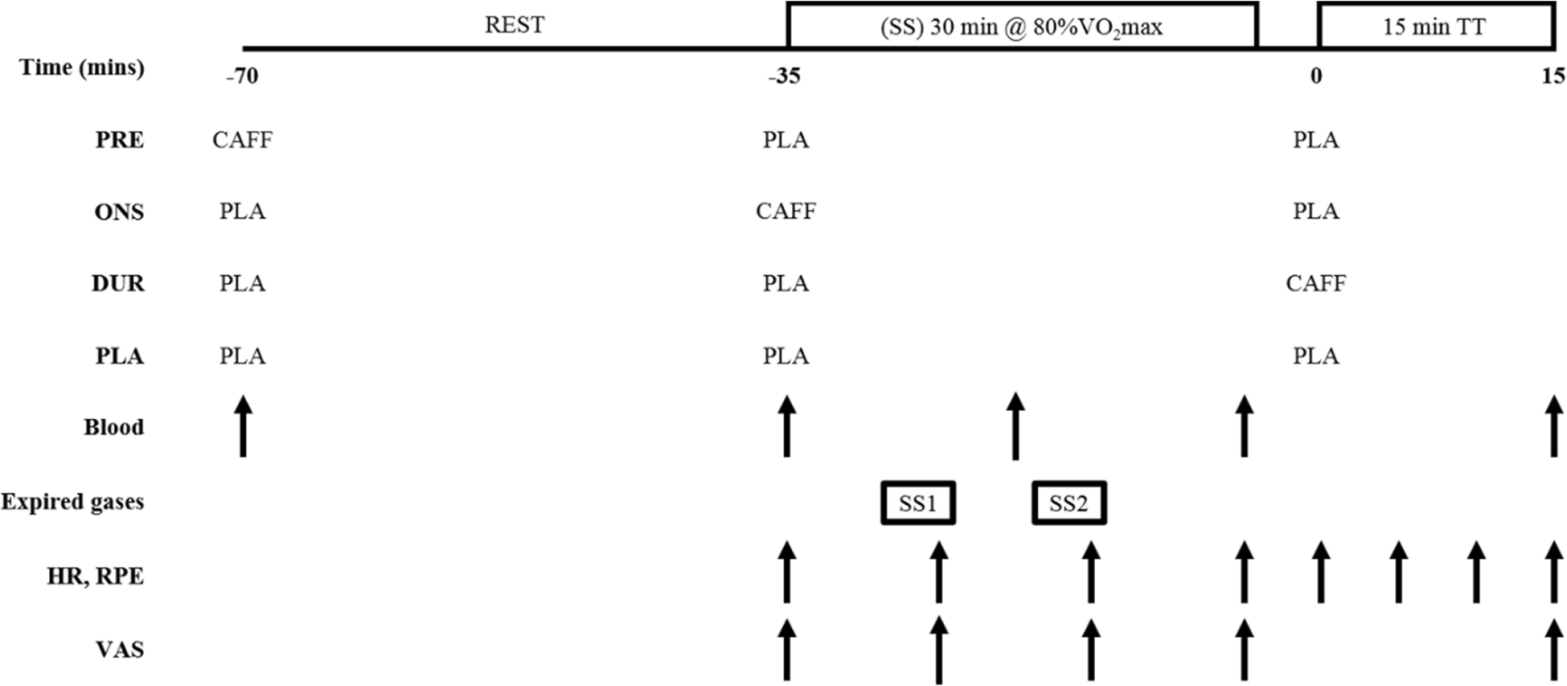
Overview of the experimental protocol. CAFF, a commercially available beverage containing 200 mg of caffeine; PLA, placebo drink; HR, heart rate; RPE, rating of perceived exertion; VAS, visual analogue scales for alertness and paraesthesia. Treatments were randomly assigned in a crossover design

Participants were randomly assigned a trial order in a double-blinded four-treatment crossover design, with each trial separated by a period of ≥ 7 days. On their first visit, participants performed an incremental cycling test to determine maximal oxygen consumption (V̇O_2max_). The second visit was a familiarization of the experimental trials, and visits 3–6 were experimental trials. Each of the 4 experimental trials consisted of participants resting for 35 min before performing 30 min of steady-state (SS) cycling at a workload aimed at eliciting 80% V̇O_2max_, preceding a standardized 5-min rest period and a 15-min time trial (TT) (Fig. 1). During the initial 35-min rest period, participants completed visual analogue scales and consumed a test drink. In order to maximize the validity of findings, we employed an exercise protocol similar to one previously employed (45-min pre-load at 70% *W*_max_ followed by a TT), where the coefficient of variation of the TT was reported as 3.5% [17]. On three of the experimental trials, participants consumed a 473 mL drink either 35 min before SS exercise (PRE), at the onset of SS (ONS), or immediately before the time trial (DUR) phases, with a 473 mL placebo drink consumed at the other two time points (i.e. three drinks per visit). The other visit (PLA) consisted of consuming the placebo drink at all three time points. The commercially available drink contained 200 mg of caffeine (~ 2.8 mg kg bm^− 1^, range 2.4 to 3.4 mg kg bm^− 1^), 1600 mg of β-alanine, 1000 mg of quercetin and 4.5 g sucrose (Beachbody Performance Energize, Beachbody LLC, USA). The placebo was taste-, colour- and calorie-matched and also contained 4.5 g sucrose.

### Pre-experimental tests

Two weeks prior to the first experimental visit, participants completed a continuous incremental V̇O_2max_ test on an electronically braked cycle ergometer (Lode Excalibur Sport, Lode, The Netherlands). Following a self-selected warm-up, participants began cycling at a power output of 150 W. The resistance was then increased by 40 W every 3 min until volitional exhaustion. Throughout the duration of the test, participants wore a face mask attached to a metabolic cart (Jaeger Oxycon Pro, CareFusion, Switzerland). V̇O_2max_ was determined as the highest 15-s average. Following a 10-min rest period, participants were familiarized with the 15-min TT protocol.

One week prior to their first experimental visit, participants were familiarized with the experimental protocol (Fig. 1). During the SS cycle, V̇O_2_ was measured repeatedly to help adjust and establish the workload required to elicit 80% V̇O_2max_. Participants could drink water ad libitum throughout the SS cycle. Bike position was noted at the end of the familiarization trial and then replicated for all experimental visits.

### Experimental trials

Participants reported to the laboratory following an overnight fast at the same time for each trial, before providing a pre-trial mid-stream urine sample for analysis of urine osmolality (Pocket Osmocheck, Vitech Scientific, UK), and body mass was measured (SECA 780, Germany) (Pre-Ex). A cannula was then inserted into an antecubital vein which was kept patent by a 0.9% sodium chloride drip (~ 1.8 mL min^− 1^). A sample of blood was drawn, immediately followed by the consumption of the first experimental drink. Participants then rested for 35 min before heart rate (Polar, Finland); alertness and paraesthesia were recorded. Alertness and paraesthesia were measured by participants marking a single line on separate, 20 cm, visual analogue scales. A second experimental drink was then consumed before the commencement of cycling at 80% V̇O_2max_ for 30 min and an immediate recording of rating of perceived exertion (RPE). Further measures of heart rate, RPE, alertness and paraesthesia were recorded at 10-min intervals throughout the SS cycle. Blood samples were taken immediately prior to the second experimental drink and at subsequent 15-min intervals during the SS cycle. A final blood sample was taken at the end of the TT. For two 5-min periods (SS1 and SS2), expired gases were recorded using a metabolic cart to determine substrate oxidation rates. After cycling for 30 min, participants had a 5-min rest period, before consuming their third experimental drink, and immediately commencing a 15-min TT. The aim of the TT was to perform as much work as possible. Heart rate and RPE were recorded at 5-min intervals during the TT. Measures of alertness and paraesthesia and a final blood sample were taken at the end of the TT. After the TT, body mass was recorded, before another urine sample was provided. There were no differences in laboratory temperature (18.4 ± 2.1 °C) or humidity (48 ± 7%) between trials, and participants were fan-cooled whilst cycling.

### Dietary and exercise standardisation

In the 24 h preceding the familiarization trial, participants were instructed to either rest or train lightly and eat a high-carbohydrate diet. Meals and training sessions were selected individually by the participants, dependent on their preferred pre-race routine, but were approved by the researchers. Participants were instructed to follow the same diet and exercise practices before each trial, and these were confirmed with the use of training and nutrition diaries. Participants were asked to disclose the use of any performance-enhancing supplements or drugs. Only participants who were not using any ergogenic supplements were included in the study. Furthermore, to standardize pre-trial plasma caffeine concentrations, participants were instructed to avoid caffeinated products for 18 h before the experimental trials.

### Blood sampling and analysis

One mL of whole blood was immediately analysed for glucose and lactate (YSI 2300 Stat Plus, USA). The remaining blood was placed into a lithium heparin tube containing 50 μL of EGTA glutathione, which was subsequently centrifuged for 10 min (4000×*g*, 4 °C). Samples were frozen immediately at − 20 °C, before being transferred to a − 80 °C freezer. Plasma caffeine, paraxanthine, theobromine and theophylline concentrations were quantified via high-performance liquid chromatography [18]. The ratio of paraxanthine to caffeine (PX:CA) at the end of the PRE trial, i.e. when the concentration of both metabolites was at a steady state, was calculated as an index of *CYP1A2* activity.

### Genotyping

Saliva samples were collected on a separate lab visit at the end of all physiological tests using the Oragene ON-600kit (DNA Genotek, Ottawa, Ontario, Canada) for DNA isolation using manufacturers recommended techniques. A StepOnePlus Real-Time PCR System (Thermo Fisher Scientific, USA) was used for the genotyping of the rs762551 SNP in the *CYP1A2* gene.

### Calculations

Maximum work capacity (*W*_max_) was calculated using the following equation: *W*_*max*_ (*W*) = *W*′ + (*40 · t*/*180*) (where *W*′ is the highest workload completed, and *t* (s) is the time attained in the final, incomplete, stage at exhaustion during the incremental exercise test). Substrate oxidation rates were calculated using stoichiometric eqs [19].

### Statistical analysis

TT performance was analysed using a repeated-measures one-way ANOVA. Data passed the Shapiro-Wilk test of normality. All other data (blood and plasma metabolites, substrate oxidation, alertness, paraesthesia, heart rate, RPE, urine osmolality and body mass) were analysed by repeated-measures two-way ANOVAs. The two-way ANOVAs performed on blood lactate and glucose, and plasma metabolites were run separately for SS and TT periods. Specific differences were identified using Tukey’s multiple comparisons post hoc tests. Effect sizes were determined by Cohen’s d. Statistical analyses were performed using GraphPad Prism 7 (GraphPad Software, Inc., USA). All data are presented as mean ± SEM, with *P* < 0.05 indicating statistical significance.

## Results

### Descriptive results

Following analysis of plasma caffeine concentrations, 1 female subject was excluded from all analyses due to very high (> 7 μmol L^− 1^) baseline concentrations. Data for plasma and blood metabolites are presented for *n* = 12 following blood-sampling problems in one trial. Of the 13 participants, 7 were homozygous for the A allele (AA), none were homozygous for the C allele (CC) and 6 were heterozygous (AC). Participants arrived at the laboratory with a similar body mass (71.0 ± 1.1 kg) and urine osmolality (586 ± 37 mOsmo kg^− 1^) for each trial. Participants were infused intravenously with 164 ± 5 mL of 0.9% sodium chloride during each trial. Following the trial, body mass remained unchanged (71.4 ± 1.1 kg), but urine osmolality decreased (432 ± 26 mOsmo kg^− 1^) (*P* < 0.0001). Two out of 13 participants identified the PLA trial. In line with what would be expected from a successfully blinded randomized trial, timing of drink administration was correctly identified in 12 of the 39 trials (PRE = 5, ONS = 5, DUR = 2). Of the non-correctly identified trials, 20 were unidentifiable, and 18 were incorrectly guessed.

### Time trial performance

**Fig. 2:**
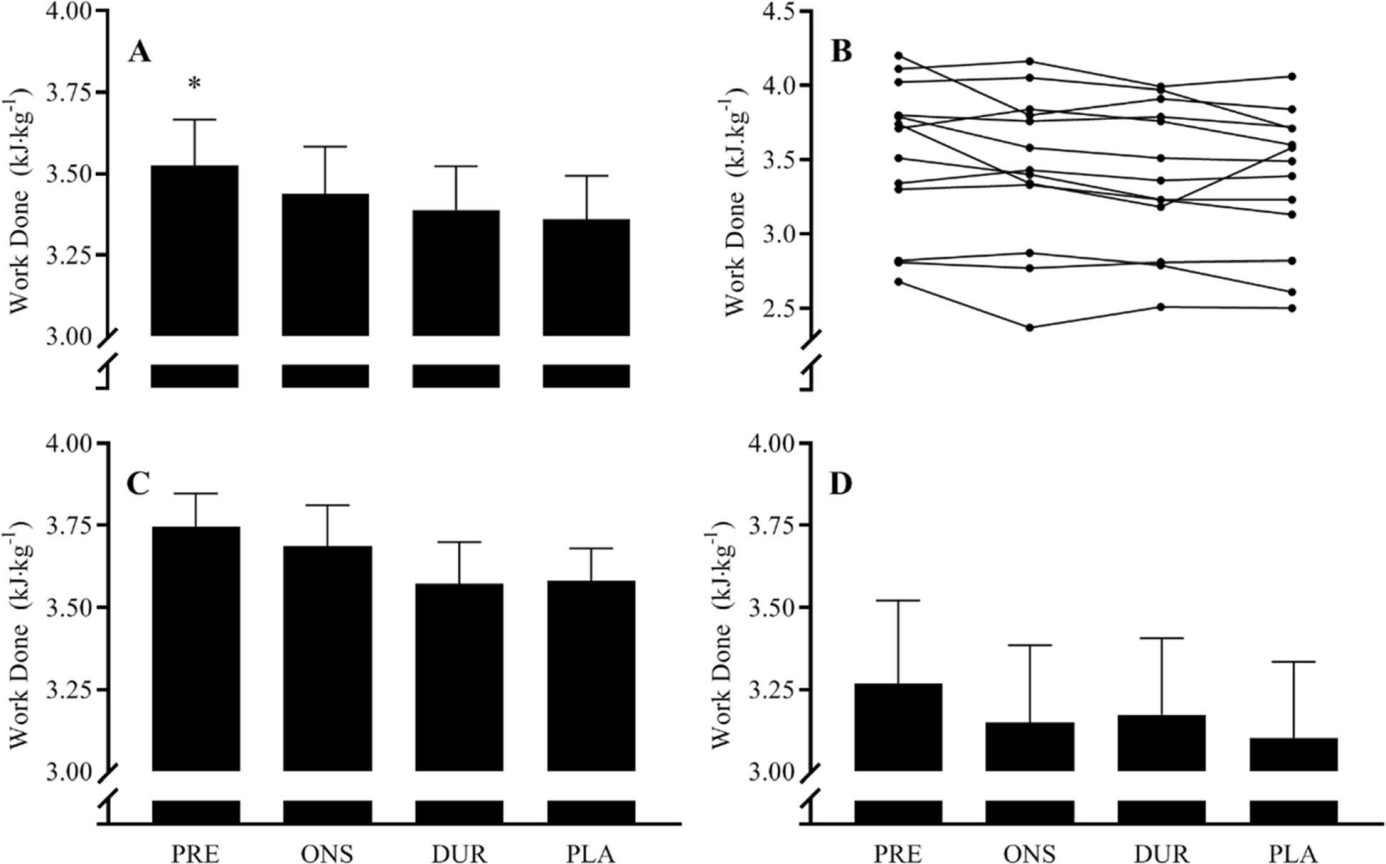
**a** Mean work done per kilogramme of body mass during a 15-min cycling time trial in the PLA, DUR, ONS and PRE trials. Data are mean for all participants ± SEM. **b** Individuals participants’ work done per kilogramme of body mass during a 15-min cycling time trial in the PLA, DUR, ONS and PRE trials. *P < 0.01, significantly different from PLA. Mean work done per kilogramme of body mass during a 15-min cycling time trial in the PLA, DUR, ONS and PRE trials for **c** AA and **d** AC genotypes

There was a significant main effect for the timing of drink ingestion on 15-min TT performance (*P* = 0.0082). The average work done was 5% higher in PRE (3.53 ± 0.14 kJ kg bm^− 1^) than PLA (3.36 ± 0.13 kJ kg bm^− 1^, *P* = 0.025, *d* = 0.35) (Fig. 2a, b). There was no significant difference between PLA and ONS (3.44 ± 0.14 kJ kg bm^− 1^, *P* = 0.3619, *d* = 0.17) or DUR (3.39 ± 0.14 kJ kg bm^− 1^, *P* = 0.925, *d* = 0.06), and no significant differences between ONS, DUR or PLA. The average power output for each of the trials were PLA 3.73 ± .015 W kg bm^− 1^, DUR 3.76 ± .015 W kg bm^− 1^, ONS 3.82 ± 0.16 W kg bm^− 1^ and PRE 3.92 ± 0.16 W kg bm^− 1^. There were no differences in performance improvement versus PLA between genotypes in PRE (Fig. 2c, d) (AA 4.7 ± 1.5%, AC 5.4 ± 1.5%, *P* = 0.771), ONS (AA 2.9 ± 1.9%, AC 1.7 ± 2.3%, *P* = 0.678) or DUR (AA − 0.26 ± 2.2%, AC 2.4 ± 1.1%, *P* = 0.323).

### 30-min steady-state cycle

**Fig. 3:**
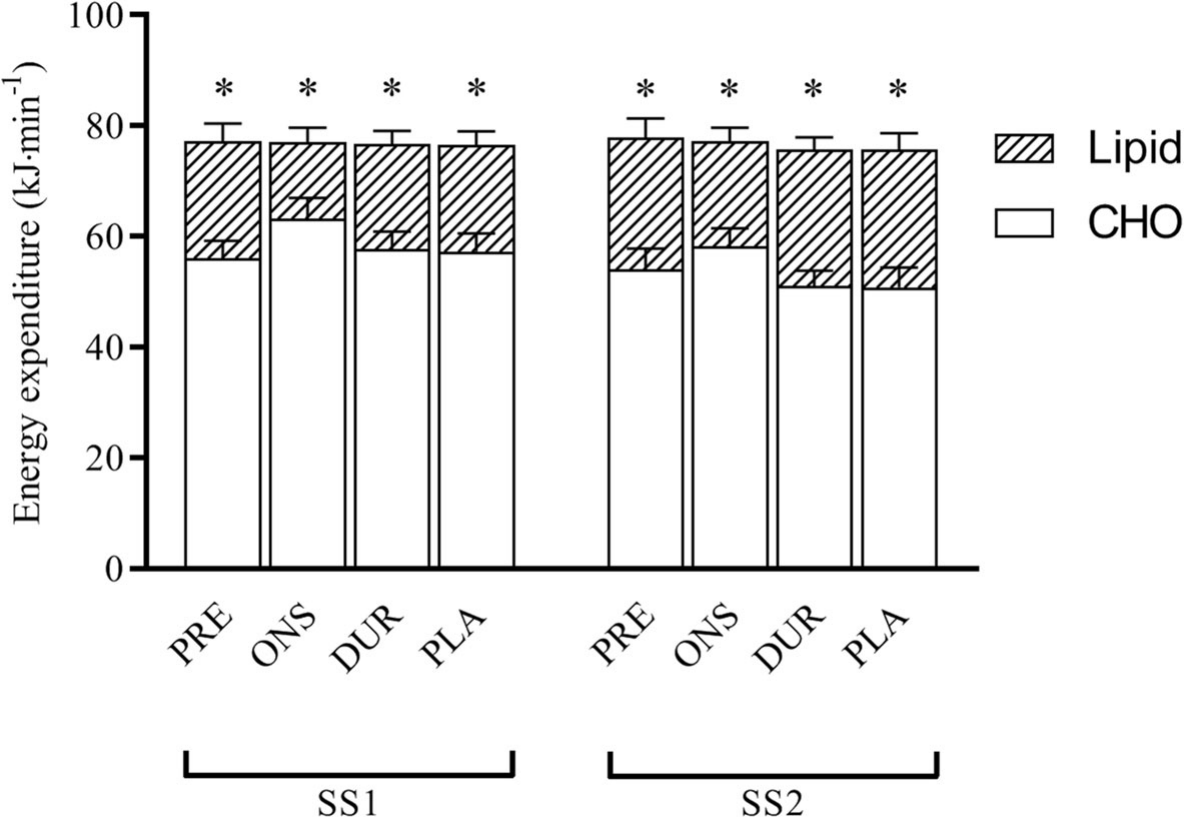
Calculated carbohydrate (CHO) and lipid (Lipid) oxidation during 30 min of SS cycling at 83 ± 1% VO2max in the PLA, DUR, ONS and PRE trials. Expired gases were measured for two periods of 5 min (SS1 and SS2)

The average power output during the SS cycle was 243 ± 8 W, which corresponded to 83 ± 1% V̇O_2max_. There was no change in relative VO_2max_ from SS1 to SS2. There was no difference between conditions in calculated carbohydrate or lipid oxidation (Fig. 3). There was, however, an effect of time, as rates of carbohydrate oxidation dropped from 58.6 ± 1.6 kJ min^− 1^ to 53.5 ± 1.7 kJ min^− 1^ (*P* < 0.0001), and lipid oxidation increased from 18.3 ± 1.4 to 23.2 ± 1.4 kJ min^− 1^ during SS1 and SS2, respectively (*P* < 0.0001).

### Blood and plasma metabolites

**Fig. 4:**
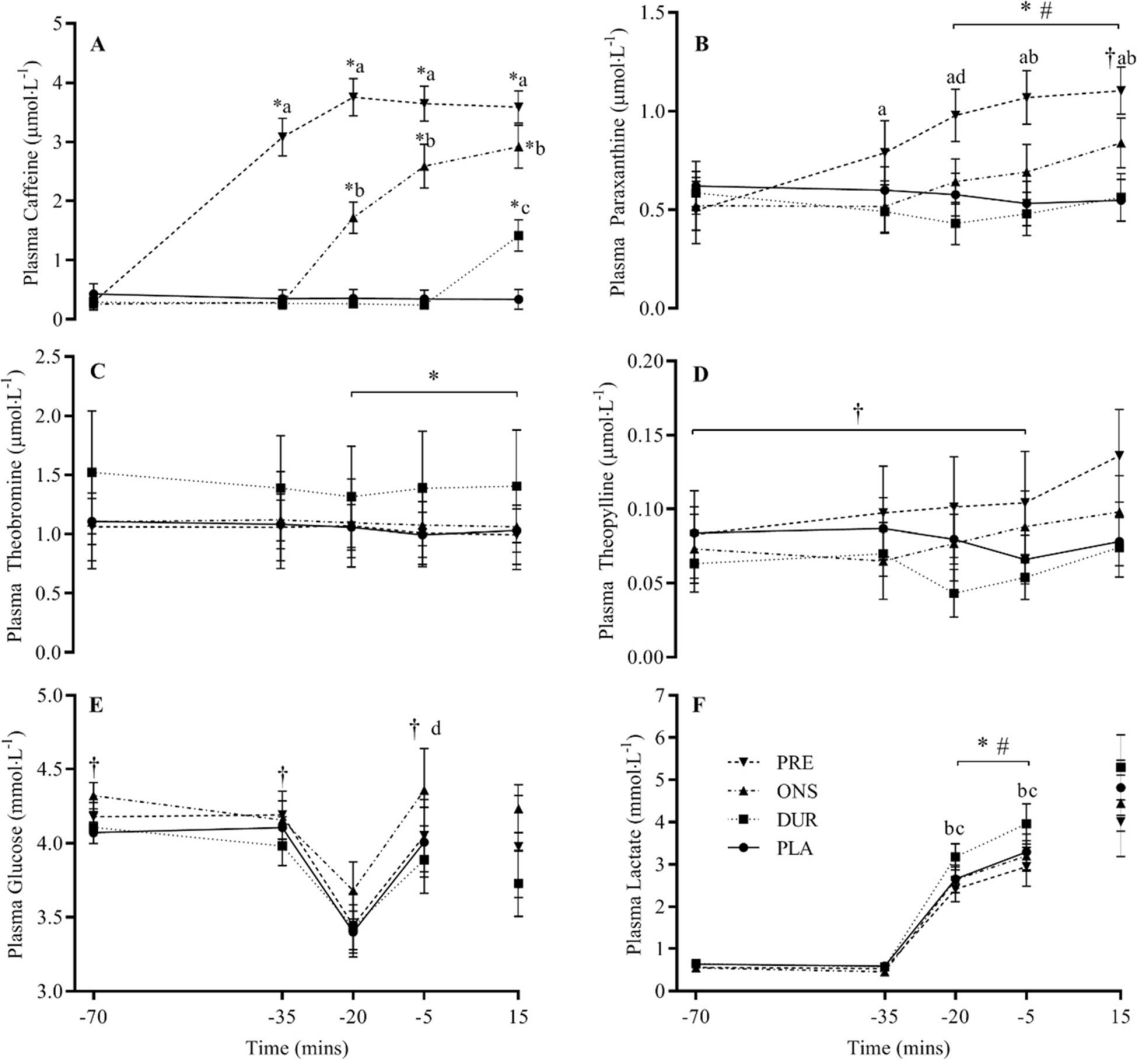
Plasma caffeine (**a**), paraxanthine (**b**), theobromine (**c**), theophylline (**d**), glucose (**e**) and lactate (**f**) during the PLA, DUR, ONS and PRE trials. Data are mean for all participants ± SEM. Main effect for time: *, significant difference from − 70; #, significant difference from − 35; †, significant difference from − 20. Main effect for condition: (a) PRE significantly different to ONS, PRE and PLA; (b) ONS significantly different to DUR and PLA; (c) DUR significantly different to PLA and (d) DUR significantly different to DUR and PLA

There were no differences in plasma caffeine concentrations between conditions at baseline (Fig. 4a). Plasma caffeine concentrations increased in all experimental conditions following ingestion of the drink *(P* < 0.0001), and there was a significant interaction effect, with peak values observed at different time points (*P* < 0.0001). Plasma paraxanthine concentrations were the same for all conditions at baseline but increased over the duration of the trial (*P* < 0.0001, Fig. 4b). There was also a main effect for condition (*P* = 0.0343), with paraxanthine concentrations higher in PRE than all other conditions from − 35 onwards and in ONS than DUR and PLA from − 20 onwards. There were no differences between conditions in plasma theobromine concentrations (*P* = 0.7498, Fig. 4c) or plasma theophylline concentrations (*P* = 0.4467, Fig. 4d). There was no difference between genotypes in PX:CA at the end of TT in PRE, i.e. when the concentration of both metabolites was at a steady state (AA 0.27, AC 0.32, *P* = 0.17, *d* = 0.95). There was no correlation between PX:CA and performance improvement in PRE vs. PLA (*r*^2^ = 0.001, *P* = 0.9112).

There were no differences between conditions at baseline, or at the start of the SS cycle, in blood glucose concentrations, however, there was a significant effect for condition (*P* = 0.0353), with glucose levels in DUR significantly lower than ONS at the end of the SS cycle (Fig. 4e). Blood glucose levels dipped significantly mid-way through the SS cycle (*P* = 0.0037), before recovering to pre-exercise levels by the end of SS.

Blood lactate concentrations rose throughout the SS cycle (*P* < 0.0001) (Fig. 4f). Concentrations were significantly higher in ONS compared to DUR and PLA but not PRE (*P* = 0.033). During the TT, there was no significant main effect for time or condition.

### Rating of perceived exertion

**Fig. 5:**
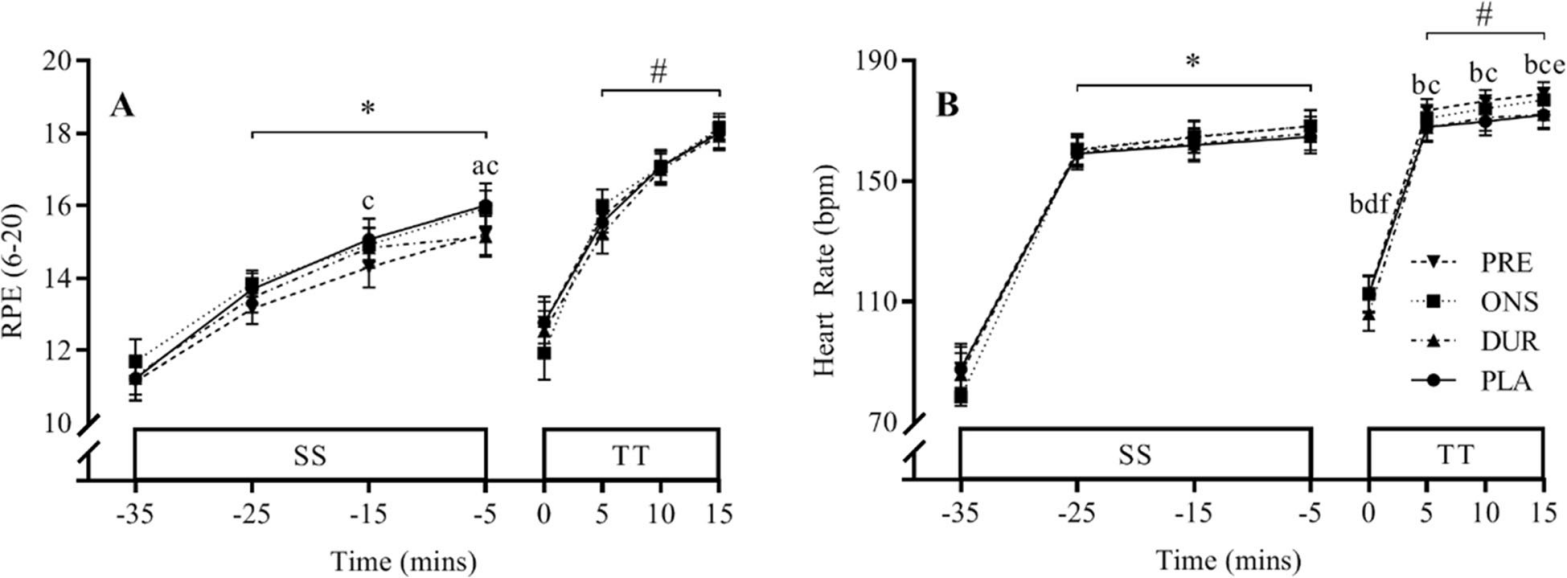
**a** RPE and **b** heart rate during 30 min of SS cycling at 80% VO_2max_ and a 15-min time trial in the PLA, DUR, ONS and PRE conditions. Data are mean for all participants ± SEM. Main effect for time: *, significant difference from − 35; #, significant difference from 0. Main effect for condition: (a) significant difference between PRE and ONS, (b) significant difference between PRE and DUR, (c) significant difference between PRE and PLA, (d) significant difference between ONS and DUR, (e) significant difference between ONS and PLA and (f) significant difference between DUR and PLA

During the SS cycle, there was a significant main effect for condition (*P* = 0.011), with max RPE in PRE (13.5 ± 0.9) significantly lower than PLA and DUR (14 ± 1 and 14.1 ± 0.9, respectively) (Fig. 5a). There was no significant difference between PRE and ONS (13.7 ± 0.9). RPE increased in all trials during the TT (*P* < 0.0001), but there was no difference between conditions.

### Heart rate

Heart rate increased significantly during the SS cycle, to a peak of 167 ± 1 beats min^− 1^ by the end (*P* < 0.0001), but there were no differences between conditions (Fig. 5b). Whilst heart rate also increased significantly during the TT for all conditions (*P* < 0.0001), there were significant differences between conditions (*P* = 0.002) in line with the amount of work performed, with the peak heart rate in PRE (179 ± 4 beats min^− 1^) exceeding those in ONS (177 ± 4 beats min^− 1^), DUR (172 ± 5 beats min^− 1^) and PLA (172 ± 4 beats min^− 1^).

### Alertness and Paraesthesia

**Fig. 6:**
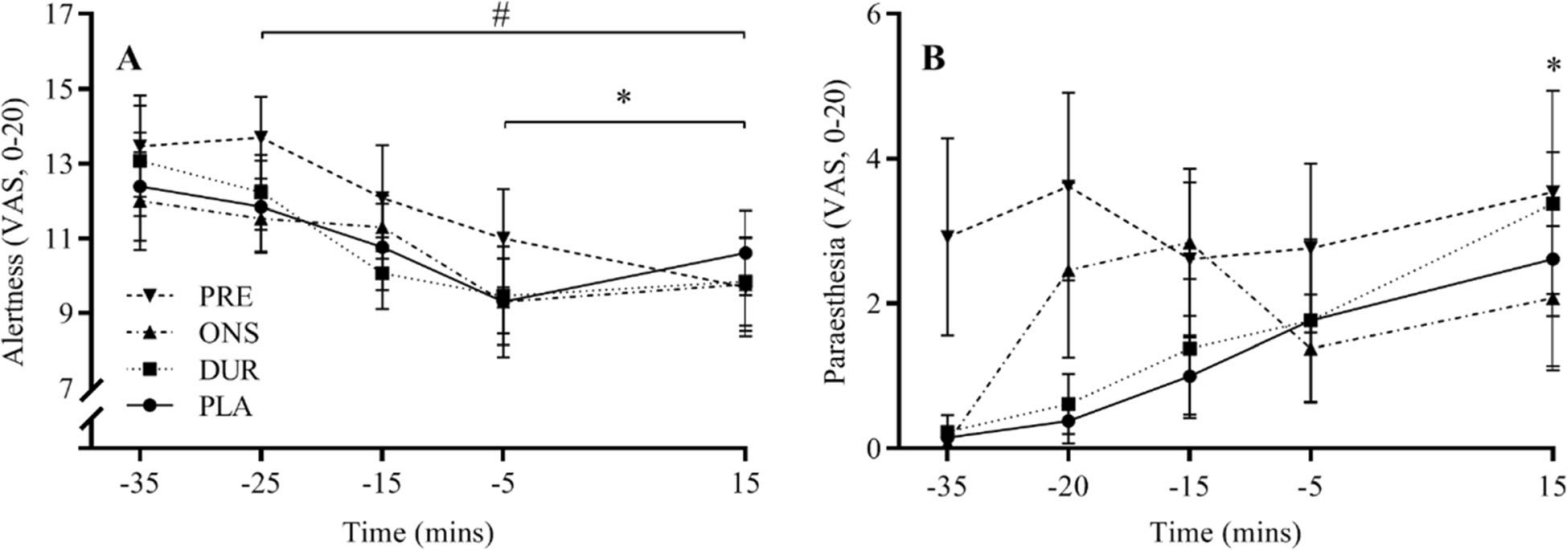
Alertness (**a**) and paraesthesia (**b**) during the PLA, DUR, ONS and PRE trials. Data are mean for all participants ± SEM. *, significant difference from − 25; #, significant difference from − 35

There were no differences between conditions for either alertness or paraesthesia throughout the trial (Fig. 6). However, alertness decreased for all conditions throughout the SS cycle (*P* < 0.0001).

## Discussion

The present study assessed the optimal timing of ingestion of a low (200 mg, 2.8 ± 0.1 mg kg bm^− 1^) dose caffeine supplement, on perception of effort, plasma caffeine metabolites and exercise performance. Cycling performance was improved by 5% following the ingestion of the supplement during the PRE condition (i.e. around 30 min prior to beginning an exercise protocol consisting of SS cycling at 80% V̇O_2max_, rest and an all-out time trial). Despite an apparent time response, the ingestion of the supplement at the onset (ONS) of, and during (DUR), the exercise protocol had no effect on performance. The lack of effect here was surprising given that the ingestion of the supplement in ONS also reduced perceived exertion during the SS exercise compared with placebo. Intriguingly, whilst plasma caffeine concentration was elevated prior to and during the TT with all supplement timing strategies, paraxanthine was only elevated prior to the TT when the supplement was ingested in the PRE condition, the only strategy to observe an ergogenic effect. It is important to note that the supplement also contained quercetin and β-alanine, and it is possible that they may have also contributed to the ergogenic effect by a synergistic or additive mechanism. However, there was no effect on performance when quercetin and β-alanine were ingested at other time points in the present study; previous findings suggest that acute supplementation of β-alanine has no effect on endurance performance, even in instances where athletes are aware of the ingestion of a supplement [20], and a recent meta-analysis concludes that chronic supplementation of quercetin has only ‘between trivial and small’ effects on performance [21]. Thus, the acute effect on performance in the current study was likely driven by caffeine as opposed to the other ingredients.

The literature suggests there is no single optimal dose of caffeine to improve performance. Whilst there seems to be little doubt that high doses (> 6 mg kg bm^− 1^) of caffeine do not provide any greater ergogenic effect compared with lower doses (1.5 to 3 mg kg bm^− 1^), with concomitant detrimental side effects also reported [7, 22], researchers have concluded that ~ 3 mg kg bm^− 1^ is the crucial optimal dose required to improve performance [1]. Indeed, supplementing with 1.5, 2 or 3 mg kg bm^− 1^ caffeine around 60 min before exercise improved cycling TT performance similarly [23, 24]. However, neither a 1.5 nor 3 mg kg bm^− 1^ dose of caffeine 180 min prior to a 7 kJ kg bm^− 1^ TT improved performance [25]. It would appear, therefore, that despite plasma caffeine concentrations remaining elevated for several hours after ingestion, there is an optimal time for ingesting low doses (1.5 to 3 mg kg bm^− 1^) of caffeine before a TT, suggesting elevated plasma caffeine concentrations per se may not be the only causative mechanism. Thus, in line with the findings that the ingestion of 2.9 mg kg bm^− 1^ caffeine around 55 min prior to the last 15 min of a cycling TT improved performance by over 7% [23], and the ingestion of either 2 or 3 mg kg bm^− 1^ caffeine 80 min before a 15 min cycling TT improved performance by 3 and 4%, respectively [24], we demonstrate that ingesting a supplement containing 200 mg (2.8 mg kg bm^− 1^) caffeine 70 min before a 15 min cycling TT improved performance by 5%. Low-dose caffeine ingestion both 35 min and, in contrast to the typical advice, 5 min before the final 15 min of a TT has also been shown to improve performance but only by ~ 2% [26, 27]; however, these findings were not replicated in the present study. Considering a low dose of caffeine 35 and 0 min before a 15 min cycling TT failed to improve performance in the present study, we can be more prescriptive and suggest that an optimal time to ingest caffeine in order to enhance performance is around 60 min before the end (i.e. last 15 min) of exercise, where perceived effort is likely to be extremely hard.

It has been proposed that inter-individual differences in genotype may alter the efficacy of caffeine supplementation [25]. Indeed, recent work has shown striking differences in the ergogenic efficacy of supplementing with caffeine around 65 min before the last 15 min of a TT, depending upon the genotype of the *CYP1A2* gene [12]. It was demonstrated that 2 or 4 mg kg bm^− 1^ caffeine improved 10-km cycling TT performance by 5 and 7%, respectively, in those with the AA genotype, had no effect on those with the AC genotype and reduced performance by 14% in those with the CC genotype. Conversely, in the present study, whilst no participants had the CC genotype, there was no difference in *CYP1A2* enzyme activity between those with the AA and AC genotypes, as shown by similar PX:CA ratios in PRE, and consequently, both groups found caffeine to be equally efficacious at improving exercise performance. Differences between findings could be due to the contrasting cycling abilities of participants recruited across studies, with previous research employing relatively untrained individuals (V̇O_2max_ 47.7 mL kg min^− 1^) [12] compared to the present study (V̇O_2max_ 64.5 mL kg min^− 1^). It is known that regular exercise increases *CYP1A2* expression [28], and it seems that the ‘slow metabolizing’ AC-carrying athletes may be able to overcome any genetic disadvantage with training to increase *CYP1A2* activity sufficiently. Whilst the results of the present study should be viewed with caution due to the relatively small sample size, our findings are in line with previous studies that have found that, in large samples sizes, there is no effect for any *CYP1A2* single-nucleotide polymorphism or haplotype on caffeine metabolism, other than in smokers with the AA genotype [29, 30]. Alternatively, the additional ingredients of the supplement (β-alanine and quercetin) may have affected caffeine metabolism.

Following the ingestion of each supplement, plasma caffeine concentrations increased in all conditions, although a plateau was only attained in PRE around 50 min after caffeine ingestion. This is in contrast with previous studies, which show that following the ingestion of ~ 200 mg of caffeine, circulating caffeine concentrations peaked at between 75- and 120-min post-supplementation [26, 31]. Again, this could be due to the training status of the participants or perhaps the additional ingredients in the supplement affecting caffeine absorption. Nevertheless, this ensured that a peak in plasma caffeine concentration was achieved in PRE before the 15-min TT where performance was improved. However, in ONS, plasma caffeine concentrations were also elevated pre-TT, where no effect on performance was observed. This would suggest that either the duration for which plasma caffeine was elevated was insufficient to cross the blood brain barrier and act on adenosine receptors to affect performance, or that an increase in the primary metabolite of caffeine, paraxanthine, must also be attained. Indeed, at the start of the TT plasma paraxanthine concentrations were elevated in PRE but not ONS. Paraxanthine is a pharmacologically more potent adenosine-receptor antagonist than caffeine [15] and is thought to have additional effects on locomotor activity by increasing dopamine levels in the dorsolateral striatum [16]. This suggests that the principal mechanism for the observed performance improvements is a reduction in perceived exertion mediated by increased paraxanthine and/or a combination of paraxanthine and caffeine, particularly as there were no differences in peripheral responses during exercise. It has been previously observed that when low-dose caffeine is ingested 195 min prior to the final 15 min of a TT, it has no ergogenic effect [24]. As paraxanthine has a half-life of 3.1 h [32], this may also suggest that a threshold plasma paraxanthine concentration exists for any performance effect. Clearly, our understanding of the mechanisms of action of caffeine and paraxanthine on exercise performance requires further investigation. A recent meta-analysis has suggested that the ergogenicity of caffeine is related only to the duration of the sport; however, we believe the findings of our current study should complement, rather than juxtapose such conclusions [33]. The present study investigated the effect of timing of caffeine supplementation on a 15-min TT and found an effect of timing on its efficacy, and we recommend that if people are performing for longer durations, they may need to alter these recommendations and ingest caffeine during exercise. We suggest that future studies looking at the ergogenicity of caffeine should quantify not only plasma caffeine, but plasma paraxanthine, as standard practice.

## Limitations

Although we cannot conclusively state that the additional ingredient within the drink (β-alanine and quercetin) had no effect on performance, previous research and meta-analyses suggest that this is the case [20, 21]. A wide range of caffeinated supplements (e.g. gums, drinks, capsules) are now available to athletes. However, as the most likely method of caffeine ingestion during cycling will be by a gel or drink, we chose to administer caffeine via a commercially available drink. Additionally, we chose to administer a standard, absolute dose of caffeine, rather than one relative to body mass. The methods of caffeine delivery were chosen to mimic the manner in which supplementation is typically provided, to maximize the ecological validity of the outcomes; however, practitioners should be prudent when applying the findings of this study to other forms of caffeine administration. Whilst there may be concerns over both caffeine withdrawal and habituation, we are confident that these will have been influential in the present study, with previous research confirming that neither effect caffeine effectiveness [34, 35]. Unfortunately, the rating of perceived exertion was the only perceptual measure used in the present study to assess the potential central effects of caffeine.

## Conclusions

In summary, a supplement containing a low (200 mg, 2.8 mg kg bm^− 1^) dose of caffeine ingested 70 min prior to a 15-min cycling TT reduced perceived exertion during cycling at 80% V̇O_2max_ and improved performance in trained cyclists by 5%. Whether these effects are due to increases in circulating caffeine per se, the caffeine metabolite paraxanthine or the combination of ingredients requires further investigation. The same supplement ingested 35 or 0 min prior to a 15-min TT had no effect on perceived exertion or TT performance. Taken together with the results from other similar studies in the literature, we suggest that cyclists should ingest supplements containing low doses of caffeine (e.g. 200 mg) around 60 min prior to the onset of expected hard exercise (i.e. 60 min before the last 15 min of exercise) in order to reduce the perception of effort and maximize the improvement in performance. This is an improvement on the current broad recommendation of ingesting caffeine-containing supplements 60 min before exercise per se. Whilst we can only make conclusions around the timing of caffeine ingestion in cyclists, a recent review of meta-analyses found caffeine improved exercise in a broad range of exercise modalities, with the magnitude of effect generally greater for aerobic exercise compared to anaerobic exercise [36]. Moreover, we recommend that practitioners do not use genotyping as a tool to discourage athletes from supplementing with caffeine, partly because genotype did not affect performance in this and other studies, but also because it is difficult to distinguish normal day-to-day variation from individual responses to a supplement [37]. It appears that the timing of caffeine is a much more important variable than an individual’s genotype when considering its efficacy, particularly in young, healthy individuals, such as athletes. Whilst it is hard to mimic the reactive and dynamic nature of road cycling in a laboratory environment, we believe the use of a validated time trial test, together with the high standard of cyclists in the present study, should give practitioners confidence in applying these outcomes to highly trained endurance athletes.

## Data Availability

The datasets used and/or analysed during the current study are available from the corresponding author on reasonable request.

## Abbreviations

bm: Body mass
*CYP1A2*: Cytochrome P4501A2
DUR: Consumption of caffeine immediately before the cycling time trial
ONS: Consumption of caffeine at the onset of steady-state exercise
PLA: Placebo condition
PRE: Consumption of caffeine 35 min before the onset of steady-state exercise
PX:CA: Paraxanthine to caffeine ratio
RPE: Rating of perceived exertion
SEM: Standard error of mean
SS: Steady-state
TT: Time trial
V̇O_2max_: Maximal oxygen consumption during an incremental cycling test
*W*_max_: Maximum work capacity at during an incremental cycling test

## References

[1] Burke LM (2008). Caffeine and sports performance. Appl Physiol Nutr Metab [Internet]..

[2] Knapik JJ, Steelman RA, Hoedebecke SS, Austin KG, Farina EK, Lieberman HR (2016). Prevalence of dietary supplement use by athletes: systematic review and meta-analysis. Sport Med..

[3] Fredholm BB, Yang J, Wang Y (2017). Low, but not high, dose caffeine is a readily available probe for adenosine actions. Mol Aspects Med [Internet]..

[4] Davis JM, Zhao Z, Stock HS, Mehl KA, Buggy J, Hand GA (2003). Central nervous system effects of caffeine and adenosine on fatigue. Am J Physiol Regul Integr Comp Physiol.

[5] Goldstein ER, Ziegenfuss T, Kalman D, et al. International society of sports nutrition position stand: caffeine and performance. J Int Soc Sports Nutr. 2010;7, 5(1) Available from: https://jissn.biomedcentral.com/articles/10.1186/1550-2783-7-5. 10.1186/1550-2783-7-5 PMC2824625 20205813

[6] Southward K, Rutherfurd-Markwick KJ, Ali A (2018). The effect of acute caffeine ingestion on endurance performance: a systematic review and meta–analysis. Sport Med.

[7] Graham TE, Spriet LL (1995). Metabolic, catecholamine, and exercise performance responses to various doses of caffeine. J Appl Physiol.

[8] Pickering C, Kiely J (2018). Are the current guidelines on caffeine use in sport optimal for everyone? Inter-individual variation in caffeine ergogenicity, and a move towards personalised sports nutrition. Sport Med.

[9] Gu L, Gonzalez FJ, Kalow W, Tang BK (1992). Biotransformation of caffeine, paraxanthine, theobromine and theophylline by cDNA-expressed human CYP1A2 and CYP2E1. Pharmacogenetics.

[10] Sachse C, Brockmöller J, Bauer S, Roots I (1999). Functional significance of a C → A polymorphism in intron I of the cytochrome P450 CYP1A2 gene tested with caffeine. Br J Clin Pharmacol..

[11] Womack CJ, Saunders MJ, Bechtel MK (2012). The influence of a CYP1A2 polymorphism on the ergogenic effects of caffeine. J Int Soc Sports Nutr.

[12] Guest N, Corey P, Vescovi J, El-Sohemy A. Caffeine, CYP1A2 genotype, and endurance performance in athletes. Med Sci Sport Exerc. 2018;(February):1 Available from: http://insights.ovid.com/crossref?an = 00005768–900,000,000-96,963. 10.1249/MSS.0000000000001596 29509641

[13] Pataky MW, Womack CJ, Saunders MJ (2016). Caffeine and 3-km cycling performance: effects of mouth rinsing, genotype, and time of day. Scand J Med Sci Sport.

[14] Algrain HA, Thomas RM, Carrillo AE (2015). The effects of a polymorphism in the cytochrome P450 CYP1A2 gene on performance enhancement with caffeine in recreational cyclists. J Caffeine Res.

[15] Benowitz NL, Jacob P, Mayan H, Denaro C (1995). Sympathomimetic effects of paraxanthine and caffeine in humans. Clin Pharmacol Ther.

[16] Orrú M, Guitart X, Karcz-Kubicha M (2013). Psychostimulant pharmacological profile of paraxanthine, the main metabolite of caffeine in humans. Neuropharmacology.

[17] Jeukendrup A, Saris WH, Brouns F, Kester AD (1996). A new validated endurance performance test. Med Sci Sports Exerc.

[18] Holland DT, Godfredsen KA, Page T, Connor JD (1998). Simple high-performance liquid chromatography method for the simultaneous determination of serum caffeine and paraxanthine following rapid sample preparation. J Chromatogr B Biomed Sci Appl.

[19] Jeukendrup AE, Wallis GA (2005). Measurement of substrate oxidation during exercise by means of gas exchange measurements. Int J Sports Med [Internet]..

[20] Bellinger PM, Minahan CL (2016). Performance effects of acute β-alanine induced paresthesia in competitive cyclists. Eur J Sport Sci [Internet]..

[21] Kressler J, Millard-Stafford M, Warren GL (2011). Quercetin and endurance exercise capacity: a systematic review and meta-analysis. Med Sci Sports Exerc.

[22] Anderson ME, Bruce CR, Fraser SF (2000). Improved 2000-m rowing performance in competitive oarswomen after caffeine ingestion. Int J Sport Nutr Exerc Metab [Internet]..

[23] Talanian JL, Spriet LL (2016). Low and moderate doses of caffeine late in exercise improve performance in trained cyclists. Appl Physiol Nutr Metab.

[24] Jenkins NT, Trilk JL, Singhal A, O’Connor PJ, Cureton KJ (2008). Ergogenic effects of low doses of caffeine on cycling performance. Int J Sport Nutr Exerc Metab.

[25] Desbrow B, Barrett CM, Minahan CL, Grant GD, Leveritt MD (2009). Caffeine, cycling performance, and exogenous CHO oxidation: a dose-response study. Med Sci Sports Exerc..

[26] Kovacs EMR, Stegen JHCH, Brouns F (1998). Effect of caffeinated drinks on substrate metabolism, caffeine excretion, and performance. J Appl Physiol.

[27] Cox GR, Desbrow B, Montgomery PG (2002). Effect of different protocols of caffeine intake on metabolism and endurance performance. J Appl Physiol.

[28] Vistisen K, Poulsen HE, Loft S (1992). Foreign compound metabolism capacity in man measured from metabolites of dietary caffeine. Carcinogenesis.

[29] Ghotbi R, Christensen M, Roh H-K, Ingelman-Sundberg M, Aklillu E, Bertilsson L (2007). Comparisons of CYP1A2 genetic polymorphisms, enzyme activity and the genotype-phenotype relationship in Swedes and Koreans. Eur J Clin Pharmacol..

[30] Jiang Z, Dragin N, Jorge-Nebert LF (2006). Search for an association between the human CYP1A2 genotype and CYP1A2 metabolic phenotype. Pharmacogenet Genomics..

[31] Kamimori GH, Karyekar CS, Otterstetter R (2002). The rate of absorption and relative bioavailability of caffeine administered in chewing gum versus capsules to normal healthy volunteers. Int J Pharm.

[32] Lelo A, Birkett D, Robson R, Miners J (1986). Comparative pharmacokinetics of caffeine and its primary demethylated metabolites paraxanthine, theobromine and theophylline in man. Br J Clin Pharmacol..

[33] Shen JG, Brooks MB, Cincotta J, Manjourides JD (2019). Establishing a relationship between the effect of caffeine and duration of endurance athletic time trial events: a systematic review and meta-analysis. J Sci Med Sport.

[34] de Gonçalves L (2017). S, Painelli V de S, Yamaguchi G, et al. Dispelling the myth that habitual caffeine consumption influences the performance response to acute caffeine supplementation. J Appl Physiol.

[35] Irwin C, Desbrow B, Ellis A, O’Keeffe B, Grant G, Leveritt M (2011). Caffeine withdrawal and high-intensity endurance cycling performance. J Sports Sci..

[36] Grgic J, Grgic I, Pickering C, Schoenfeld BJ, Bishop DJ, Pedisic Z. Wake up and smell the coffee: caffeine supplementation and exercise performance - an umbrella review of 21 published meta-analyses. Br J Sports Med. 2019:1–9. 10.1136/bjsports-2018-100278 30926628

[37] Burke LM, Peeling P. Methodologies for investigating performance changes with supplement use. Int J Sport Nutr Exerc Metab. 2018:1–11 Available from: https://www.researchgate.net/publication/323346877_Methodologies_for_Investigating_Performance_Changes_With_Supplement_Use%0Ahttps://journals.humankinetics.com/doi/10.1123/ijsnem.2017-0325. 10.1123/ijsnem.2017-0325 29468949

